# The Army Canteen

**Published:** 1901-07

**Authors:** 


### THE ARMY CANTEEN.

THE abolition of the army canteen or post exchange, which was said to have been due to the efforts of the Woman's Christian Temperance Union, seems to have encountered the general disfavor of the medical profession, in and out of the army. The American Medical Association has made formal protest, and the Association of Military Surgeons of the United States Army has taken similar action. There appears to be but little doubt that the influence of the army canteen was decidedly good upon the amount and quality of the drinking done by the soldiers, and that its removal will eliminate a conservative feature of this important phase of army life. During the existence of this institution, drinking was done decently, and the quality of the malt liquor (the only kind sold) was good. As it is now, the soldier must leave the post to quench his thirst, and the amount consumed to effect this is limited only by his capacity, physical and financial, while the quality of the stuff passed to him over the bar is, to say the least, questionable. As a result of this, the soldier gets drunk and disorderly and freely exhibits his condition on the street and winds up at an establishment where virtue is easy or in the lockup, where the practice of virtue is subjected to forced cultivation. The recent shooting of a policeman by a drunken private from the Fort McPherson Barracks was, in all likelihood, a direct result of the abolition of the canteen and the reformatory efforts of the W. C. T. U. The general condition of the prohibition question is such that radical measures are scarcely feasible from an economic standpoint. More good is accomplished by judicious restriction than by summary abolition. It would seem wiser to reinstate the canteen, and it is hoped that the several protesting medical bodies will cause Congress to reconsider its rash and ill-judged action, and will follow the views of sanitarians and medical men rather than those of women with more heart than head.

---

DEATH OF AN EMINENT FOREIGN PROFESSOR.—Joseph Fodor, M.D., professor of hygiene at the University of Budapest, has recently died. He was born in 1843, studied under Pettenkofer at Munich, and later under Baron Liebig. Dr. Fodor was, after his master Pettenkofer, the best known of the European sanitarians, and did much toward rendering Budapest the healthy and beautiful city it now is. He was a man of many gifts, and was for some time joint editor of the medical journal, *Orvosi Hetilap*.—*New York Medical Record.*

